# Oncological Patient Anxiety in Imaging Studies: the PET/CT Example

**DOI:** 10.1007/s13187-016-1069-3

**Published:** 2016-07-02

**Authors:** Carla Abreu, Ana Grilo, Filipa Lucena, Elisabete Carolino

**Affiliations:** 1 King’s College London & Guy’s and St. Thomas’ PET Centre, St. Thomas’ Hospital, Lambeth Palace Road, SE1 7EH, London, UK; 2 Department of Social Sciences and Humanities, Scientific Area of Psychology, Higher School of Health Technology, Polytechnical Institute of Lisbon, Portugal, Av. Dom João II Lote 4.69.01, 1990-096 Lisbon, Portugal; 3 Scientific Area of Nuclear Medicine, Higher School of Health Technology, Polytechnical Institute of Lisbon, Portugal, Av. Dom João II Lote 4.69.01, 1990-096 Lisbon, Portugal; 4 Department of Natural and Exact Sciences, Area of Mathematics, Higher School of Health Technology, Polytechnical Institute of Lisbon, Portugal, Av. Dom João II Lote 4.69.01, 1990-096 Lisbon, Portugal

**Keywords:** Anxiety, Positron emission tomography, Patient perspective, Oncology

## Abstract

The purpose of this study was to investigate the subjective perception of anxiety pre- and post-procedure, and explore the relationship between demographic, clinical variables and cancer patients’ anxiety during a positron emission tomography/computed tomography (PET/CT) scan. Two hundred and thirty-two oncological out patients, with clinical indication for performing an ^18^F-2-fluoro-2-deoxy-D-glucose (^18^F-FDG) PET/CT scan and attending a nuclear medicine (NM) department, participated in the study. Patients’ anxiety and subjective experience of PET/CT were examined using two self-report questionnaires. The pre-procedure questionnaire focused on demographic information, level of knowledge regarding the scan and subjective perception of anxiety before the procedure. The post-procedure questionnaire included the subjective perception anxiety after the procedure, information adequacy and satisfaction with the NM department. The self-reported data indicate that patients were anxious during PET/CT. Furthermore, our data revealed a significant difference between the anxiety pre-procedure and post-procedure (*z* = −3909, *p* < 0.05), in which the anxiety pre-procedure has significantly higher values. No significant correlation was found between anxiety and age of the patients, education levels, adequacy of information or satisfaction with the NM Department. Perception of anxiety post-procedure differs between gender (*U* = 5641, *p* = 0.033). In conclusion, PET/CT generated anxiety levels in oncological patients, especially before the procedure. Although patients seemed to be satisfied with information delivered by staff and with the NM Department, attention has to be focused on effective interventions strategies that help patients to reduce anxiety.

## Introduction

Anxiety is a complex reaction to potentially uncertain or dangerous situations, anticipating and preparing the individual to act and respond adequately [1, 2]. It is often associated with physical symptoms such as muscle tension, fatigue, tremors and frequent urination. Cancer patients commonly experience emotional distress, including anxiety, as a response to the initial diagnosis and also throughout the course of the disease [3, 4], usually linked to fears related to the illness, such as pain, loss of social and professional roles, separation, uncertainty towards the future and eventually, death [1, 2].

Medical imaging technologies and techniques have evolved greatly in the last decade, with marked improvements in the diagnosis and follow-up of diseases, especially in cancer. Positron emission tomography/computed tomography (PET/CT) with ^18^F-2-fluoro-2-deoxy-D-glucose (^18^F-FDG) is a less invasive tool available for the assessment of patients with suspected cancer [2, 5]. The use of ^18^F-FDG, an analogue of glucose, contributes to the assessment of glucose metabolism, which is increased in most cancers [5, 6], thus making this a sensitive and useful imaging modality for diagnosis, staging and restaging and assessing the response to therapy of oncological patients [5]. PET/CT is a vital examination among many tests that oncological patients are subjected to.

In this context, medical imaging can be perceived by cancer patients as a threat, causing fear and anxiety, as it may confirm the diagnosis or change the therapeutic approach [2]. Also, discomfort and claustrophobia are reported by patients, probably due to the confrontation with intimidating and unfamiliar technology and more specifically, the confined nature of the acquisition procedure [7, 8].

When performing a PET/CT with ^18^F-FDG, extensive and diverse information is communicated to the patient. Prior to the scan day, a brief and simple explanation of the procedure should be given and also information regarding fasting times, hydration and exercise. On the day of the scan, obtaining a pertinent patient history and clear explanation is of utmost importance to obtain image quality and diagnostic accuracy, provide a comfortable and as minimal invasive experience as possible for the patient [9]. However, high anxiety can affect the patient’s experience and interfere with the workflow of the nuclear medicine (NM) department (e.g. amount of extra time spent to tranquillise the patient or the presence of movement-related artefacts eventually requiring image repetition) [5, 8]. Additionally, the change in physiologic parameters caused by anxiety may affect the normal biodistribution of ^18^F-FDG (e.g. uptake of ^18^F-FDG in muscles or brown adipose tissue) and interfere with the accurate results of the PET/CT [8, 9].

The purpose of this study was to investigate the subjective perception of anxiety pre- and post-procedure, and explore the relationship between demographic, clinical variables and cancer patients’ anxiety during a PET/CT scan.

## Material and Methods

### Participants

This cross-sectional prospective study was based on the population of oncological patients with clinical indication to perform PET/CT with ^18^F-FDG and who were 18 years old or older.

A non-probabilistic sample included all consecutive oncological patients who were scheduled for a PET/CT with ^18^F-FDG in the NM department of a private clinic of Lisbon (Portugal), between April 2014 and July 2014. All the patients who participated in this study answered all the questions on both questionnaires.

Exclusion criteria included patients aged under 18 years old, visibly incapacitated, unable to understand the content of the questionnaires and who had not totally filled in the questionnaires. No restrictions were made as to cancer stage or point in the process of treatment.

Prior to the study, authorisation from the department’s administration board was obtained. The aim of the study was orally explained to all patients and confidentiality and anonymity were assured. All participants signed an informed consent form. From the patients initially recruited, 14 (6 %) refused to participate in the study and therefore the final sample included 232 patients.

### Procedure

A member of the healthcare team made initial contact with the patient and introduced the study. Patients who agreed to participate were asked to complete the questionnaires, after the consent form was signed.

The patients filled in two self-report questionnaires at two different moments of the PET/CT scan: on arrival at the NM department and prior to any information from the Allied health professionals (AHP) (pre-procedure) and immediately after the end of the image acquisition (post-procedure), while in the NM department. Patients received paper copies of the two questionnaires. The PET/CT scan was performed on a Siemens Biograph 16 PET/CT scanner; 90 min after an intravenous injection of 296–370 MBq (8–10 mCi) of ^18^F-FDG. The scan was acquired from the base of the skull to the mid-thigh. The acquisition time was 3 min/bed with total scanning time being approximately 20 min.

### Data Collection

Two questionnaires were developed involving a review of the literature to identify the problems and needs of oncological patients performing imaging scans. A pre-test was performed in two steps: firstly, a draught of the questionnaires was reviewed by the AHP team for a preliminary content validation and secondly, the questionnaires were administered to ten patients undergoing PET/CT scan to point out potential problems and misunderstandings. Based on the feedback provided by the pre-test, some questions were reworded and others eliminated. The Portuguese version of the questionnaires was used for the data collection.

The pre-procedure questionnaire comprised questions related to demographic information (e.g. age, gender and literacy) and the level of knowledge regarding the scan. The subjective perception of pre-procedure anxiety was also assessed through a ten-point Likert scale, previously used for this purpose in other NM studies [10].

The post-procedure questionnaire explored the patient’s experiences during the procedure and the performance of the AHP. Close-ended questions assessed the AHP performance regarding the communication of information, as well as the patient need to seek further information prior to the scan. A ten-point Likert scale was used for the assessment of the subjective perception of anxiety, satisfaction with care and satisfaction with the information provided.

### Data Analysis

Normality was tested by using the Kolmogorov-Smirnov test. The difference between the subjective perception of anxiety pre-procedure and post-procedure was examined for statistical significance using the Wilcoxon test.

The Spearman correlation coefficient test was applied to study the relationship between age, adequacy of information provided by the professional and satisfaction with the subjective perception of anxiety pre-procedure as well as post-procedure.

Mann-Whitney test was used to compare subjective perception of anxiety pre-procedure and post-procedure between gender, prior PET/CT, whether there was any autonomous research for information and information about the PET/CT provided by AHP. In addition to these tests, the Kruskal-Wallis test was used to compare the subjective perception of anxiety pre- and post-procedure between the four educational groups and the four options indicating the reasons for a PET/CT, because the normality assumption was also not verified.

Data were analysed using SPSS version 22.0 for Windows. The differences were considered statistically significant at a *p* value less than 0.05.

## Results

The sample consisted of 232 patients; male and female patients were almost equally represented. The mean age of the patients included was of 61 ± 13.4 years (62.8 ± 13.1 years for male and 59.4 ± 13.5 years for female) and are mostly educated with secondary education (59.9 %) (Table 1).

**Table 1:** Characteristics of study patients and mean (SD) on information provided, global satisfaction and subjective perception of anxiety perceived by patients

Variable	Mean ± SD	Number (*N*)	Percentage (%)
Gender			
Male Female		113	48.7
		119	51.3
Age	61 ± 13.4	232	
Education			
Without studies Basic education (6–15 years) Secondary education (15–17 years) Higher education (17 years+)		12	5.2
		50	21.6
		139	59.9
		31	13.4
First PET/CT			
Yes No		165	71.1
		67	28.9
Reason for PET/CT			
Initial staging Characterisation Assess response to treatment Exclude recurrence Does not know		93	49.7
		27	14.4
		35	18.7
		32	17.1
		45	19.4
Autonomous information seeking			
Yes No		72	31.0
		160	69.0
Information provided by AHP Yes No			
		171 61	73.7 26.3
Adequacy of information provided by the professionals	7.3 ± 2.2	232	
Global satisfaction with NM Department	6.8 ± 2.4	232	
Anxiety pre-procedure	6.4 ± 2.7	232	
Anxiety post-procedure	5.7 ± 2.6232		

One hundred and sixty-five of the 232 patients (71.1 %) underwent the scan for the first time. A total of 80.6 % knew the reason to perform the PET/CT. The most frequently reported reason to perform the PET/CT was the “initial staging” of oncological disease (49.7 %). The majority of the patients (69 %) reported not having searched for information prior to the scan. Of the 72 patients that searched for information prior to the scan, 54.5 % reported “radiation effects” as the main predisposing concern factor. 73.7 % of the patients received information related to the PET/CT procedure by the AHP. The adequacy of information provided by the professionals presented a mean value of 7.3 ± 2.2. The overall mean satisfaction with the department was 6.8 ± 2.4 (Table 1).

The mean level of anxiety perceived by the patient prior to the procedure was 6.4 ± 2.7 and post-procedure was 5.7 ± 2.6. There was a statistically significant difference between the anxiety pre-procedure and post-procedure (*z* = −3909, *p* = 0.000) in which the anxiety post-procedure has significantly lower values.

From the analysis of the variables that could affect the subjective perception of anxiety pre-procedure and post-procedure, no significant correlation was found with the age of the patients, adequacy of information provided or global satisfaction as shown in Table 2.

**Table 2:** Correlation between age, adequacy of information provided and global satisfaction on subjective perception of anxiety

	Anxiety	
	Pre-procedure *ρ* (*p* value)	Post-procedure *ρ* (*p* value)
Age (global) Age (male) Age (female)	0.033 (0.618) 0.058 (0.545) −0.016 (0.863)	−0.040 (0.542) −0.042 (0.659) −0.078 (0.3999)
Adequacy of information provided by the professionals	−0.025 (0.742)	−0.032 (0.675)
Global satisfaction	−0.058 (0.381)	−0.037 (0.578)

Significance was determined using Spearman’s correlation coefficient (*p* value statistically significant at <0.05)

The relationship between patients that underwent the scan for the first time and subjective perception of anxiety pre- and post-procedure was not significant (*U* = 4657.5, *p* = 0.058; *U* = 4743.5, *p* = 0.088, respectively).

In relation to gender, there was no significant difference between men and women in the subjective perception of anxiety before the procedure (*U* = 5803.5, *p* = 0.07). However, the subjective perception of anxiety post-procedure differs significantly between men and women (*U* = 5641, *p* = 0.033), with men presenting higher values.

No statistically significant differences were found in subjective perception of anxiety pre-procedure and reason to perform the PET/CT scan (K-W = 0.857, *p* = 0.836) and education levels (K-W = 0.984, *p* = 0.805). No such significant difference exists in subjective perception of anxiety post-procedure and reason to perform the PET/CT scan (K-W = 2.303, *p* = 0.512) and education levels (K-W = 1.299, *p* = 0.729).

The relationship between patients that searched for information prior to the scan and subjective perception of anxiety pre-procedure was not significant (*U* = 5679.5, *p* = 0.864), neither was significantly different the subjective perception of anxiety post-procedure (*U* = 4918.5, *p* = 0.073).

## Discussion

The present study provided detailed understanding of the anxiety levels during a PET/CT scan. Our results indicate that cancer patients experience anxiety during the PET/CT scan. Anxiety levels were higher prior to the scan (6.4 ± 2.7), probably reflecting initial anxiety related to the scan procedure and environment. Post scan anxiety levels were significantly lower (5.7 ± 2.6), suggesting that a considerable part of the anxiety felt by the patient is related with the imaging procedure itself. Lledó et al. [10]*,* in a research with patients that underwent conventional NM scans, obtained a perception of anxiety result of 4.3 while the procedure was being carried out. The average levels of anxiety in our study are clearly higher than those found by Lledó et al. [10], whether before or after the scan. This difference may be related with the inclusion criteria of both studies: in our study only oncological patients were included whereas in Lledó et al. [10] patients were randomly included from all the NM procedures, suggesting that oncological patients are exposed to higher levels of anxiety. In fact, our results are in accordance with the literature [11], showing that the majority of the patients performed the PET/CT scan for initial staging of an oncological disease and, for the remaining patients, the reasons for performing the PECT/CT scan were directly related with the progression of the disease. In both cases, the outcome of the scan will provide information regarding the disease and so, anxiety appears as a response to the uncertainty towards this result [1, 2]. Additionally, the anxiety results are above the midpoint on the Likert scale and, therefore, should not be disregarded as it is known that high anxiety levels affect ^18^F-FDG biodistribution (e.g. uptake of ^18^F-FDG in muscles or brown adipose tissue), interfering with the accurate results of the PET/CT. Also, considering that muscular tension associated with anxiety can cause pain and discomfort in a scan like PET/CT [12], it is important to emphasise anxiety as a relevant factor for patient safety.

Despite being the first PET/CT for 71.1 % of the subjects, we found no statistically significant differences in the subjective perception of anxiety between these patients and those who had already undergone PET/CT previously. Previous research has demonstrated that even patients who had undergone PET/CT previously also experience high levels of anxiety, which was thought to be related with an anticipatory effect regarding its possible negative result [2]. Further, the anxiety levels of patients who underwent PET/CT for the first time could be related to the lack of knowledge about the procedure, possible side effects and its result [2]. With reference to the reason behind performing the scan, it was established that the majority of the patients’ scans were carried out in the context of initial staging of an oncology disease. Considering that almost two thirds of the patients in our study performed the scan for their first time, a possible justification for the high pre-procedure anxiety levels is the fear of the unknown regarding the procedure and the effects of radiation. Although the levels of anxiety decrease post-procedure, these should not be ignored.

Although almost 20 % of patients included in our study reported not receiving any information before the procedure, the information provided by the professionals was felt to be adequate (7.3 ± 2.2). This seems to be an unusual result since the patient’s lack of understanding of a PET/CT with ^18^F-FDG and conventional NM is a recurring theme in literature. According to Nightingale et al. [13], during the administration of a cardiac SPECT/CT, some patients at the NM department appeared to have an incomplete understanding of the procedures, some of which seemed surprised during the explanation of such procedures. The lack of understanding in relation to the scan was also previously identified by Groves et al. [14]*,* who noticed patients were astounded by the complexity of the scan and were consequently poorly prepared for it. On the other hand, our results disagree with what Domènech et al. [1] found, who reported that the quality of information influences the perception of anxiety.

From our results, 69 % of the patients admitted not having looked up information relevant to the scan before undergoing the procedure. In agreement with De Man et al. [15], it is possible that these patients avoided researching information as a coping strategy. In addition, the result attained foresees that the majority of patients count on the AHP as the main source of information, which highlights the essential role they have in preparing for the scan. In this way, the AHP during the initial contact with the patient should assess whether there has been prior research for information and whether the patient needs any clarification. It is common knowledge that a negative effect can be caused by anxiety on clinical outcomes and the general patient experience. The execution of a diagnostic scan such as a PET/CT, where the information transmitted is extensive, requires effective communication that allows the patient to understand and memorise the information as well as anticipating emotional reactions. In a scan with the PET/CT characteristics, the absence of information may be considered serious as it could jeopardise the patient’s safety and the quality of the scan.

Very few studies evaluating prior awareness of PET/CT scan were found when reviewing the literature [16, 17]. Similarly to our study, the results obtained by Andersson et al. [16] show that, despite 26 % of the patients not having a prior knowledge of PET/CT, the image quality was not affected.

In accordance with what Pifarré et al. [2] found in their PET-CT study, in our research, it was found that men evince greater anxiety than women (*U* = 5641, *p* = 0.033). More investigation is needed to further explore the difference in anxiety between gender, as this is not a common result in other studies [13, 18].

The insight to the treatment being received at the NM department is fundamental for patient satisfaction. The degree of global satisfaction with the NM department was 6.8 ± 2.4. In fact, 61.4 % of patients felt that they had the chance to explain their concerns to an AHP, who is also reported by Andersson et al. [16] who found that 65–70 % of the patients were satisfied with the interaction with the nursing staff, their communication abilities and professional skills. Although the satisfaction in our study was high, Reyes-Perez [19] obtained a higher satisfaction level (8.96). According to Lledó et al. [10], patients that have the chance to explain themselves feel more satisfied.

Considering the levels of anxiety both pre- and post-procedure found in our study, it suggests that AHP should focus on implementing specific strategies to reduce anxiety related to PET/CT, and possibly interfere with patients’ well-being and diagnostic accuracy.

Anxiety reduction strategies, like the following, have been proved to be valuable in this regard. For information improvement, Andersson et al. [20] suggest a website with information about PET/CT procedures to increase patient knowledge before the scan. Information should be understandable and not include details about radiation, risks factors and potential adverse reactions of intravenous radiopharmaceutical since these matters increase the patient’s anxiety level [20]. Furthermore, information needs to be complemented with positive interaction (trust and reassurance) between AHP and patient. Acuff et al. [21] found that making patients aware that they can contact the AHP during imaging helps to lower their anxiety. Apart from using audiovisual imagery in the PET/CT uptake room [8], which has proved to lower patient anxiety, the use of music as environmental distraction may also be used as a coping strategy for the patient [21]. Habituation in a PET/CT simulator before the scan [22], a visit to the control room before imaging, a device to signal for adjustment of music volume and a clock visible during scan procedure [20, 23] are also relatively simple changes that have been introduced with success in MR imaging and could be used in PET/CT. Further research including these strategies would make possible to investigate the association between oncological patients referred for staging and anxiety.

### Limitations

The current study has some limitations. Two questionnaires were used to collect data in the present study. Some of the questions were constructed by the authors for this particular study and had not been tested before with regard to validity and reliability, which limits the possibility of internal validity of the present study. However, the response rate was high and the low number of unanswered questionnaires indicates that the patients found the questionnaires relevant and easy to understand. Being one of the first investigations on patients’ experiences of PET/CT scan and an exploratory study, it contributes with relevant findings to this unexplored area of research.

## Conclusion

When performing PET/CT scans, oncological patients perceive a high level of anxiety, independently of the clinical reason for having the examination. The information related with the procedure provided by healthcare professionals was found to be an important factor in decreasing the overall perception of anxiety, and therefore it should be used as a strategy for coping, especially in the pre-procedure phase, where the anxiety levels are higher. Additionally, male patients were found to have higher levels of anxiety and strategies should be implemented specifically for this group of patients. The goal is to obtain PET/CT quality images while making the patient experience as non-invasive as possible. In fact, by decreasing the patient’s anxiety, the AHP can improve patients’ comfort and their overall experience.
